# Rapid modification of antibodies on the surface of liposomes composed of high-affinity protein A-conjugated phospholipid for selective drug delivery

**DOI:** 10.1016/j.bbrep.2021.101067

**Published:** 2021-07-02

**Authors:** Susumu Hama, Mika Sakai, Shoko Itakura, Eiji Majima, Kentaro Kogure

**Affiliations:** a Research Institute of Pharmaceutical Sciences, Faculty of Pharmacy, Musashino University, Tokyo, 202-8585, Japan; b Department of Biophysical Chemistry, Kyoto Pharmaceutical University, Kyoto, 607-8414, Japan; c ProteNova Co. Ltd., Kagawa, 769-2604, Japan; d Institute of Biomedical Sciences, Tokushima University Graduate School, Tokushima, 770-8505, Japan

**Keywords:** Immuno-liposomes, Drug delivery, Antibody, Protein A, Cancer

## Abstract

Antibody-modified liposomes, immuno-liposomes, can selectively deliver encapsulated drug ‘cargos’ to cells via the interaction of cell surface proteins with antibodies. However, chemical modification of both the antibodies and phospholipids is required for the preparation of immuno-liposomes for each target protein using conventional methods, which is time-consuming. In the present study, we demonstrated that high-affinity protein A- (Protein A-R28: PAR28) displaying liposomes prepared by the post-insertion of PAR28-conjugated phospholipid through polyethylene glycol (PEG)-linkers (PAR28-PEG-lipo) can undergo rapid modification of antibodies on their surface, and the liposomes can be delivered to cells based on their modified antibodies. Anti-CD147 and anti-CD31 antibodies could be modified with PAR28-PEG-lipo within 1 h, and each liposome was specifically taken up by CD147- and CD31-positive cells, respectively. The cellular amounts of doxorubicin delivered by anti-CD147 antibody-modified PAR28-PEG-lipo were significantly higher than those of isotype control antibody-modified liposomes. PAR28-PEG-lipo can easily and rapidly undergo modification of various antibodies on their surface, which then makes them capable of selective drug delivery dependent on the antibodies.

## Highlights

• PAR28-PEG-lipo underwent rapid modification of antibodies on their surface.
• PAR28-PEG-lipo were modified with various antibodies with Fc regions.
• Antibody-modified PAR28-PEG-lipo were taken up by the targeting cells.
• Antibody-modified PAR28-PEG-lipo delivered DXR into the targeting cells.

## Graphical abstract

**Figure:**
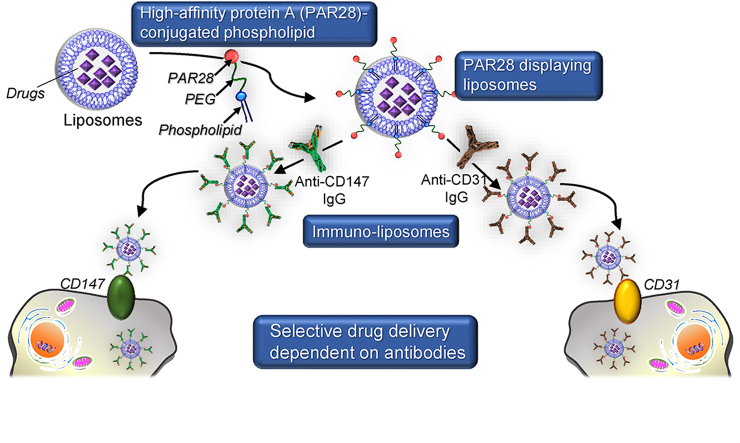

## 1 Introduction

In cancer chemotherapy, one drug has been used for the treatment of various types of cancer. For example, Doxorubicin (DXR) is the most widely used anti-cancer drug globally, and the administration of DXR alone or in combination with other drugs is used to treat various malignancies including breast, ovarian and lung cancer, neuroblastoma, and lymphoma [1]. Since liposomal DXR was approved by the Food and Drug Administration (FDA) in 1995, its application has been expanded so as to avoid serious side effects such as cardiotoxicity [2]. Liposomal DXR, Doxil ®, is polyethylene-glycol (PEG)-modified liposomes encapsulating DXR, and is passively delivered to tumors via enhanced permeability and retention (EPR) effects by using the high blood circulation potency of PEG [3]. Although many passive targeting nanoparticles exploiting strategies similar to Doxil ® have been developed [3], it has been reported that such nanoparticles exhibit lower delivery efficiency to target sites than active targeting ones [4].

For the active targeting of nanoparticles, cell-specific ligands are modified on the surface of nanoparticles to specifically bind with complementary proteins on cell surfaces [5]. Among various ligands, antibodies represent an excellent means of active targeting from the viewpoint of their high affinity for target proteins and stability in blood, and antibody-drug conjugates (ADC) are very much endorsed by the FDA [6]. However, the clinical application of drugs conjugated to antibodies is limited due to their solubility and degradability in the bloodstream, and because of the difficulty of site-directed conjugation of antibodies [7].

To overcome these obstacles, antibody-modified liposomes, immuno-liposomes, are anticipated as an active targeting drug delivery system [8]. Liposomes are bilayer vesicles composed of phospholipids and can encapsulate various drugs, including hydrophobic and hydrophilic drugs and nucleic acids. Because the antibodies are modified on the surface of liposomes, drugs encapsulated inside the liposomes can be selectively delivered to targeted cells without the conjugation of antibody to drugs. In conventional methods for the preparation of immuno-liposomes, antibodies are covalently conjugated to phospholipids in liposomes [9]. To prevent the antibody binding site from being masked by conjugation with phospholipids, site-directed conjugation of antibody to phospholipids is a main strategy. For conjugation, antibody is chemically modified with amino, thiol, imine or aldehyde groups, and phospholipids are also modified with corresponding reactive groups [9]. Such chemical modification decreases the binding affinity of antibody and will affect the physicochemical properties of liposomes. In addition, chemical conjugation is needed for every target protein for the preparation of each immuno-liposome, which is time-consuming and hampers clinical applications of antibody-based targeted therapy, even though various useful antibodies have been developed.

Therefore, we focused on protein A, which can potently bind with the crystallizable fragment (Fc) region of immunoglobulin G (IgG) for the development of immuno-liposomes being capable of rapid modification of IgG [10]. Namely, displaying protein A on the surface of liposomes should make it possible to prepare immuno-liposomes by simple incubation with various antibodies. However, it is known that protein A has low affinity for certain IgG isotypes [10]. Moreover, the stability of protein A is relatively low under the alkaline conditions necessary for effective amino-coupling reactions in which some proteins are conjugated to phospholipids [10]. Therefore, the protein A derivative PAR28, which has potent affinity for various IgG isotypes and has high stability in alkaline conditions, has been previously developed [11].

Herein, we show that PAR28-displaying liposomes composed of PAR28-conjugated phospholipids via PEG linkers can simply modify antibodies on the surface of liposomes. To evaluate cell-specific delivery depending on antibodies modified with the liposomes, in this study, we used anti-human CD147 antibody and anti-human CD31 antibodies, which are both IgG1 isotypes derived from mouse, and which show low affinity for protein A. CD147, known as extracellular matrix metalloproteinase inducer (EMMPRIN), is widely expressed on the surface of melanoma cancer cells, whose overexpression contributes to cell proliferation, invasion, and metastasis [12]. CD31, known as platelet-endothelial cell adhesion molecule (PECAM), is ubiquitously expressed on the endothelium of vasculature and is a surface marker for vascular endothelial cells [13]. Here, we show that liposomes modified with anti-CD147 antibody and anti-CD31 antibody via PAR28 are specifically taken up by CD147-positive human melanoma cell line A375 and CD31-positive human endothelial cell line HUEhT-2, respectively. Furthermore, we show that DXR encapsulated in liposomes modified with anti-CD147 antibody is effectively delivered into A375 cells.

## 2 Materials and methods

### 2.1 Materials

NIH3T3 cells, a mouse fibroblast cell line, were obtained from RIKEN BRC Cell Bank (Wako, Japan). A375 cells, a human melanoma cell line, and HUEhT-2 cells, an immortalized human umbilical vein endothelial cell line were obtained from the JCRB Cell Bank (Osaka, Japan). 3-(N-succinimidyloxyglutaryl) aminopropyl, polyethyleneglycol-carbamyl distearoylphosphatidyl-ethanolamine (DSPE-PEG-NHS), N-(Succinimidyloxy-glutaryl)-L-α-phosphatidylethanolamine, Distearoyl (DSPE-NHS) and 1,2-distearoyl-sn-glycero-3-phosphocholine (DSPC) were obtained from NOF Corporation (Tokyo, Japan). Cholesterol, dihexadecyl phosphate (DCP) and DXR hydrochloride were purchased from Sigma-Aldrich (St. Louis, MO, USA). 1,2-dioleoyl-sn-glycero-3-phosphoethanolamine-N-(lissamine rhodamine B sulfonyl) (Rh-PE) were purchased from Avanti Polar Lipid (Alabaster, AL, U.S.A.). Anti-human CD31 antibody (mouse IgG1 κ), anti-human CD147 antibody (mouse IgG1 κ) and mouse IgG1 κ isotype control were purchased from eBioscience (San Diego, CA, USA). Tetramethylrhodamine (TRITC)-labeled rabbit anti-mouse polyclonal antibody was obtained from Dako (Glostrup, Denmark).

### 2.2 Synthesis of PAR28 conjugated phospholipids

**Fig. 1:**
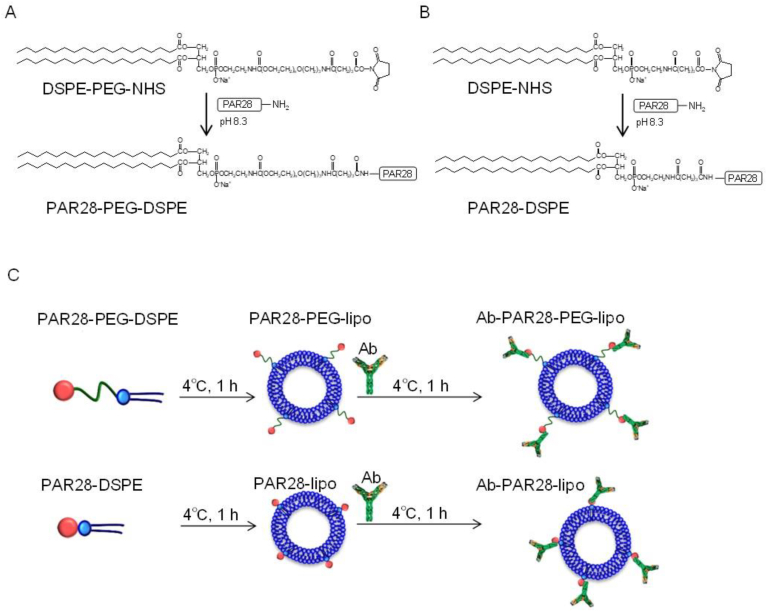
Schema of the synthesis of PAR28-phospholipids and preparation of PAR28-displaying liposomes. PAR28-PEG-DSPE (A) and PAR28-DSPE (B) were synthesized by an amine coupling method via N-hydroxysuccinimide esters (NHS) at pH 8.3. (C) Antibody (Ab) modified PAR28-PEG- and PAR28-liposomes were prepared by post-insertion of PAR28-phospholipids into anionic liposomes at 4 °C for 1 h, followed by incubation with antibody at 4 °C for 1 h.

PAR28 conjugated phospholipids were synthesized by an amine coupling method via N-hydroxysuccinimide esters (NHS) (Fig. 1A and B). Briefly, to exchange buffer, PAR28 solution was subjected to dialysis in reaction buffer (0.1 M NaHCO_3_, 0.05 M NaCl, pH 8.3) at 4 °C, and then the PAR28 solution was mixed with DSPE-PEG-NHS or DSPE-NHS at a molar ratio of 1:5 with continuous stirring. After incubation at room temperature for 1 h, 1.5 M hydroxylamine (pH 8.5) was added to stop the reaction. After further incubation at room temperature for 1 h, the mixture was subjected to dialysis in PBS at 4 °C to remove unreacted materials. The two PAR28-cojungated phospholipids, PAR28-PEG-DSPE and PAR28-DSPE, were confirmed by 4700 Proteomics Analyzer MALDI-TOF/TOF (Applied Biosystems, Foster City, CA, USA).

### 2.3 Preparation of PAR28-displaying liposomes

Anionic liposomes were prepared by a simple lipid film hydration method according to our previous report [14], and then PAR28-conjugated phospholipids were inserted into the liposomes. Briefly, DSPC and DCP dissolved in chloroform, and cholesterol dissolved in ethanol were mixed at a molar ratio of 8:2:1, and the solvent was evaporated under nitrogen gas to form a thin lipid film. The dried film was hydrated by PBS and the suspension was sonicated using a bath-type sonicator (ULTRASONIK 14B, NEY, CA, USA). To prepare PAR28-displaying liposomes, the liposomes were incubated with PAR28-PEG-DSPE or PAR28-DSPE (1 mol% of total lipids) at 4 °C for 1 h (Fig. 1C). Particle sizes and surface charges were determined by dynamic light scattering and laser doppler, respectively, using a Zetasizer nano (Malvern Ins., Ltd.). To modify antibodies on the surface of PAR28-displaying liposomes, antibodies were incubated with PAR28-displaying liposomes at 4 °C for 1 h.

### 2.4 Cell culture

NIH3T3 and A375 cells were cultivated in Dulbecco's modified Eagle's medium (DMEM) supplemented with 10% fetal bovine serum (FBS). HUEhT-2 was cultivated in MCDB131 medium (Life Technologies, Carlsbad, CA, USA) supplemented with 10% FBS, 10 mM glutamine, 5 mg/L heparin sodium and 30 mg/L endothelial cell growth supplement (BD Biosciences, Franklin Lakes, NJ, USA). The cells were incubated at 37 °C, 21% O_2_, and 5% CO_2_ in humidified conditions.

### 2.5 Analysis of cell-associated liposomes by flow cytometry

NIH3T3 cells (2 × 10^4^ cells), A375 cells (4 × 10^4^ cells) or HUEhT-2 cells (7 × 10^4^ cells) were seeded in 24-well plates. After 24 h of cultivation, cells were washed with PBS and then treated with 1 mol% Rh-PE-labeled liposomes at 5 μM lipid concentration in serum-free medium. After 2 h incubation at 37 °C, cells were collected and then washed with PBS supplemented with 2% FBS. The samples were subjected to FACScaliber flow cytometry (BD Biosciences, Franklin Lakes, NJ, USA). When cell-associated antibody was determined, cells were treated with only anti-CD147, anti-CD31 antibody, or mouse IgG1 κ isotype control at 4 °C for 1 h and then were incubated with TRITC-labeled secondary antibody at 4 °C for 1 h. The levels of cell-associated liposomes and antibodies were displayed as mean fluorescence intensity ratio (MFIR) calculated from the fluorescence intensity of the sample divided by the fluorescence intensity of control sample.

### 2.6 Observation of intracellular distribution of liposomes by confocal laser scanning microscopy

The intracellular distribution of liposomes was determined according to a previous report [15]. Briefly, A375 cells (5 × 10^3^ cells) were seeded in 96-well imaging plates (BD Biosciences) coated with 0.002% poly-l-lysine (PLL). After 24 h incubation, cells were treated with 1 mol% Rh-PE-labeled liposomes at 0.3 μM lipid concentration in serum-free medium at 37 °C for 1 h. After washing with PBS, nuclei and endosomes/lysosomes were stained with Hoechst 33342 and LysoTrackerTM Green DND-26 (Thermo Scientific, Waltham, MA, USA), respectively. The intracellular distribution of liposomes was observed by confocal laser scanning microscopy (CLSM) using an LSM 510 META instrument (Carl Zeiss Co. Ltd., Jena, Germany).

### 2.7 Intracellular delivery of DXR mediated by liposomes

DXR was encapsulated into liposomes by a remote loading method [16]. The thin lipid film prepared as described above was hydrated with 300 mM citric acid solution and then vesiculated by sonication. The liposomes were passed through a Sephadex G-50 column equilibrated with 10% sucrose and then the concentration of cholesterol in liposomes was determined by Wako Cholesterol E test kit (Wako, Oosaka, Japan). DXR solution was mixed with liposomes at a concentration of 0.2 mg/mg lipid, followed by incubation at 65 °C for 1 h. DXR-encapsulated liposomes were purified through Sephadex G-50 columns equilibrated with PBS. To determine the concentration of DXR in liposomes, the optical absorbance (490 nm) was measured following the disruption of liposomes with 1% Triton-X. To investigate the intracellular delivery of DXR, A375 cells (5 × 10^3^ cells) were seeded into 96-well imaging plates coated with PLL. After 24 h cultivation, cells were treated with DXR-encapsulated liposomes at 0.5 μM DXR concentration at 37 °C for 6 h. DXR taken up by cells was observed by CLSM. The ratio of DXR-positive cells was estimated from five individual images.

### 2.8 Statistical analysis

Statistical analysis was conducted using one-way ANOVA followed by Tukey-Kramer HSD test. P values < 0.05 were considered to be significant.

## 3 Results

### 3.1 Preparation of PAR28-displaying liposomes

For the preparation of PAR28-displaying liposomes, anionic liposomes were used as basic liposomes to avoid non-specific cellular binding via electrostatic interactions between liposomes and cells in this study [14]. When PAR28- phospholipids were displayed on the surface of liposomes, PAR28-phospholipids were post-inserted into the liposomes to avoid PAR28 denaturation by organic solvents included in the formation of thin lipid films followed by liposomalization (Fig. 1C). The physicochemical properties of liposomes are shown in Table 1. When PAR28-DSPE was post-inserted into anionic liposomes (PAR28-lipo), the particle size increased and negative surface charge was attenuated, suggesting that PAR28 was modified with liposomes. In PAR28-PEG-DSPE, PAR28 is linked with DSPE via PEG. The PEG linker will cause an increase in the flexibility of PAR28 on the surface of liposomes, contributing to effective association of antibody [17]. Furthermore, the PEG modification results in liposomal stabilization, as well as enhanced blood circulation necessary for in vivo applications [18]. As shown in Table 1, the negative surface charge of liposomes post-inserted with PAR28-PEG-DSPE (PAR28-PEG-lipo) was attenuated in comparison with that of PAR28-lipo. These results suggested that PAR28-PEG-DSPE was successfully inserted into liposomes.

**Table 1:** Particle size and ζ-potential of liposomes.

	Particle size (nm)	Surface charge (mV)
Anionic lipo	134 ± 26	-26 ± 1.9
PAR28-lipo	296 ± 70	-20 ± 3.0
PAR28-PEG-lipo	212 ± 56	-15 ± 0.9

### 3.2 Cellular uptake of PAR28-PEG-lipo modified with antibody

**Fig. 2:**
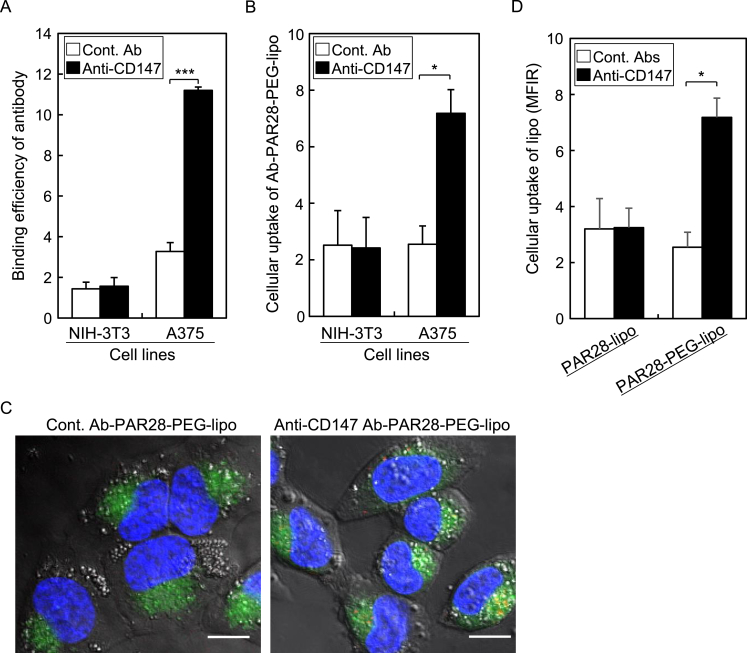
Cellular uptake and distribution of antibody-modified liposomes. (A) Binding efficiency of anti-CD147 antibody to NIH3T3 and A375 cells. (B) Cellular uptake of antibody-modified liposomes (lipo) into NIH3T3 and A375 cells. (D) Comparison of cellular uptake between PAR28-lipo and PAR28-PEG-lipo modified with antibodies in A375 cells. (A, B, D) White and black columns indicate isotype control IgG (Cont. Ab) and anti-CD147 antibody (Anti-CD147), respectively. Data show mean fluorescence intensity ratio (MFIR) of the mean fluorescence intensity of non-treated cells. n = 3, *P < 0.05, ***P < 0.001. (C) CLSM images of the intracellular distribution of isotype control IgG (Cont. Ab-PAR28-PEG-lipo) and anti-CD147 antibody-modified PAR28-PEG-lipo (Anti-CD147 Ab-PAR28-PEG-lipo). Red, green and blue signals show liposomes, endosomes/lysosomes, and nuclei, respectively. Scale bar: 10 μm. (For interpretation of the references to colour in this figure legend, the reader is referred to the Web version of this article.)

Firstly, we examined the binding efficiency of anti-CD147 antibody to NIH3T3 and A375 cells. In this study, isotype control IgG of anti-CD147 antibody was used to determine specific binding of anti-CD147 to cells. The binding efficiency of anti-CD147 antibody to A375 was significantly higher than that of isotype control, whereas anti-CD147 antibody exhibited almost no binding to NIH3T3 cells (Fig. 2A). These results indicated that A375 cells are CD147-positive, but NIH3T3 cells are not. Next, we rapidly prepared immune-liposomes by incubation of PAR28-PEG-lipo with anti-CD147 or isotype control IgG at 4 °C for 1 h and examined the cellular uptake of PAR28-PEG-lipo modified with antibody. As shown in Fig. 2B, the uptake efficiency of PAR28-PEG-lipo modified with anti-CD147 antibody was 3-fold higher than that with isotype control IgG in CD147-positive A375 cells. In CD147-negative NIH3T3 cells, cellular uptake of PAR28-PEG-lipo modified with anti-CD147 antibody was low, similar to that with isotype control IgG. These results suggested that anti-CD147 antibody displayed on the surface of PAR28-PEG-lipo via PAR28 binds with the corresponding CD147 antigen on A375 cells.

Next, we examined the cellular distribution of anti-CD147 antibody-modified PAR28-PEG-lipo in A375 cells. When anti-CD147 antibody was modified with PAR28-PEG-lipo, many fluorescent signals indicating liposomes were observed inside cells, while the modification of isotype control IgG showed no such signals (Fig. 2C). In cells treated with anti-CD147 antibody-modified PAR28-PEG-lipo, liposomes were observed as yellow dots by their colocalization with an endosome/lysosome marker. These results suggested that anti-CD147 antibody-modified PAR28-PEG-lipo was taken up by cells through CD147-associated endocytosis.

To determine the necessity of PEG linkers between PAR28 and DSPE on the cellular association of immunoliposomes, we compared the cellular uptake of PAR28-PEG-lipo and PAR28-lipo in CD147-positive A375 cells. In contrast to PAR28-PEG-lipo, the cellular uptake of PAR28-lipo with anti-CD147 antibody was at a comparable level to that of control (Fig. 2D), indicating that PAR28-lipo could not be specifically taken up by cells mediated by the interaction of anti-CD147 antibody with CD147 protein on the cell surface. Therefore, PEG linkers would be needed for the specific cellular uptake of PAR28-displaying liposomes via their modified antibodies.

### 3.3 Versatility of PAR28-PEG-lipo as a targeted carrier

**Fig. 3:**
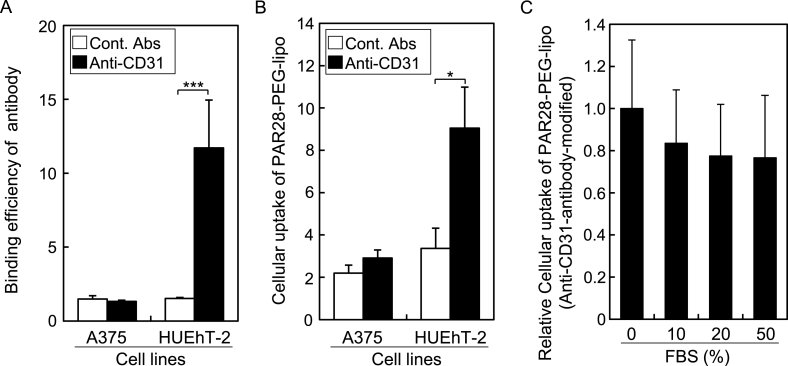
(A, B) Comparison of cellular uptake of anti-CD31 modified PAR28-PEG-lipo between CD31-negative A375 and CD31-positive HUEhT-2 cells. (A) Binding efficiency of anti-CD31 antibody. (B) Cellular uptake of antibody-modified liposomes (lipo). (A, B) White and black columns indicate isotype control IgG (Cont. Ab) and anti-CD31 antibody (Anti-CD31), respectively. (C) Effects of fetal bovine serum (FBS) on the cellular uptake of PAR28-PEG-lipo modified with anti-CD31 antibody in HUEhT-2 cells. Data show mean fluorescence intensity ratio (MFIR). n = 3, *P < 0.05, ***P < 0.001.

PAR28-PEG-lipo can quickly modify antibodies on their surface via the interaction of PAR28 with the Fc region of IgG, such that various antibodies with Fc regions will be modified with PAR28-PEG-lipo. Therefore, we prepared CD31-targeted liposomes by incubation of PAR28-PEG-lipo with anti-CD31 antibody, as shown in Fig. 1C, and examined the cellular uptake into CD31-positive HUEhT-2 cells. As shown in Fig. 3A, the binding affinity of anti-CD31 antibody used in this study was significantly higher than that of control antibody in HUEhT-2 cells. On the other hand, this anti-CD31 antibody exhibited comparable binding affinity to control antibody in A375 cells. These results suggested that this anti-CD31 antibody could specifically bind with CD31 proteins on HUEh-2 cells, and that A375 cells were CD31-negative. Correlated with Fig. 3A, the cellular uptake of PAR28-PEG-lipo modified with anti-CD31 antibody was significantly higher than that with control antibody in HUEh-2 cells, while such anti-CD31 antibody-dependent cellular uptake of PAR28-PEG-lipo was not observed in CD31-negative A375 cells. These results suggested that antibodies with Fc regions capable of binding with PAR28 can be quickly modified on the surface of PAR28-PEG-lipo, and these liposomes are taken up by cells depending on affinity of the antibody for the corresponding surface protein.

Notably, a great variety of antibodies exist in blood. If the affinity between PAR28 and antibody-modified liposomes is low, targeted antibody on the liposomes may be replaced by circulating antibody, resulting in the loss of targeted antibody-mediated cellular uptake. To exclude this undesirable possibility, we investigated the effect of serum on the cellular uptake of PAR28-PEG-lipo modified with anti CD31 antibody in HUEhT2 cells. As shown in Fig. 3C, the cellular uptake of liposomes in the presence of FBS was almost at the same level as that in the absence of FBS, even in the presence of 50% FBS. Therefore, targeted antibody on liposomes is not replaced by circulating antibody due to the high affinity of PAR28 for the targeted antibody. This suggests that PAR28-PEG-lipo modified with targeted antibody would be useful for in vivo applications as a targeted drug delivery system.

### 3.4 Targeted delivery of DXR mediated by anti-CD147 antibody-modified PAR28-PEG-lipo

**Fig. 4:**
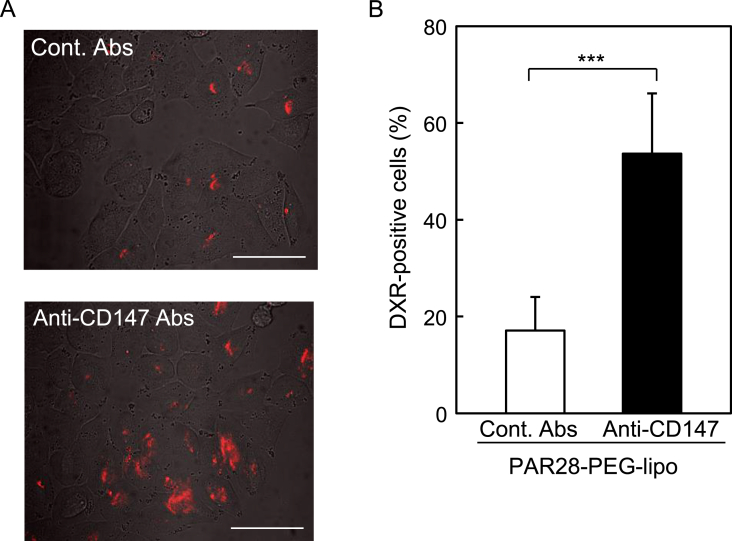
Targeted delivery of doxorubicin mediated by anti-CD147 antibody modified PAR28-PEG-lipo. CD147-positive A375 cells were treated with doxorubicin (DXR)-encapsulated PAR28-PEG-lipo modified with isotype control IgG (Cont. Ab) or anti-CD147 antibody (Anti-CD147). After incubation for 6 h, intracellular DXR was observed by CLSM. (A) Typical CLSM images of intracellular DXR delivered by Cont. Ab (upper image)- and Anti-CD147 (lower image)- modified liposomes. Red signals indicate DXR. Scale bar: 50 μm. (B) the percent of DXR-positive cells. n = 5, ***P < 0.05. (For interpretation of the references to colour in this figure legend, the reader is referred to the Web version of this article.)

To evaluate the functionality of antibody-modified PAR28-PEG-lipo as a targeted drug carrier, we determined the targeting efficiency of anti-CD147 antibody-modified PAR28-PEG-lipo encapsulating DXR. When CD147-positive A375 cells were treated with anti-CD147 antibody-modified PAR28-PEG-lipo encapsulating DXR, red fluorescent signals indicating intracellular DXR were observed in more cells than after treatment with control antibody (Fig. 4A). When DXR-positive cells were enumerated from five individual CLSM images, the percent of positive cells after treatment with anti-CD147 antibody-modified PAR28-PEG-lipo was significantly higher than that with the control (Fig. 4B). These results suggested that anti-CD147 antibody-modified PAR28-PEG-lipo could deliver DXR into CD147-positive cells.

## 4 Discussion

In the present study, we developed PAR28-PEG-lipo to modify antibodies on the surface of liposomes rapidly without the chemical modification of both the antibodies and phospholipids (Fig. 1C).

As shown in Table 1, the negative surface charge of PAR28-PEG-lipo was attenuated in comparison with that of PAR28-lipo. It has been previously reported that PEG modification attenuates surface charge on charged liposomes [19], suggesting that PAR28-PEG-lipo has a PEG layer on the surface. Furthermore, the particle size of PAR28-PEG-lipo was smaller than that of PAR28-lipo, suggesting that the PEG layer on PAR28-PEG-lipo contributes to an improvement in liposomal stability by avoiding aggregation, unlike PAR28-lipo [18].

Anti-CD147 antibody could be modified with PAR28-PEG-lipo within 1 h (Fig. 1C), and the liposomes were specifically taken up by CD147-positive cells through CD147-associated endocytosis (Fig. 2B and C). CD147 expressed on cell surfaces is a clathrin-independent endocytosis (CIE) cargo protein, and is translocated to tubular endosomes following internalization [20,21]. Thus, CD147 does not translocate to lysosomes, unlike the clathrin-dependent endocytosis cargo protein, transferrin receptor [22]. Ligand-modified liposomes often have a common cellular uptake mechanism involving surface proteins binding with ligands on liposomes [23]. Therefore, the result of Fig. 2C suggested that anti-CD147 antibody-modified PAR28-PEG-lipo might be taken up by cells through the CIE pathway, and be localized in tubular endosomes, not in late endosomes and lysosomes.

As shown in Fig. 2D, PAR28-lipo without PEG-linker could not be specifically taken up by cells mediated by the interaction of anti-CD147 antibody with CD147 protein on the cell surface. This result suggested that PEG linkers would enhance the flexibility of antibody modified with PAR28-PEG-lipo, leading to specific binding of antibody to targeted surface proteins, followed by specific cellular uptake. Recently, predictive simulations of chemical and biological system have been used to understand the ligands-binding to target proteins and structural stability of liposomes [24,25]. Such theoretical calculations would be a useful tool for the rational design of PAR28-displaying liposomes.

As shown in Fig. 4B, the DXR-positive cells treated with the anti-CD147 antibody-modified PAR28-PEG-lipo encapsulating DXR was significantly higher than that with the control. The DXR-targeting efficiency value, which was calculated from the percent of DXR-positive cells by anti-CD147 antibody-modified PAR28-PEG-lipo and dividing that by the control value, as in Fig. 4B, was approximately 3.1, which was similar to the uptake efficiency of anti-CD147 antibody-modified PAR28-PEG-lipo without DXR (Fig. 2B). These results suggested that intracellular DXR, observed in Fig. 4A, can be delivered by antibody-modified PAR28-PEG-lipo encapsulating DXR following the cellular uptake of liposomes themselves.

In addition to liposomes, attractive systems such as a metal-organic framework have been developed for drug delivery [26,27]. Since PAR28 can rapidly modify antibodies on the surface of drug carriers, PAR28-mediated modification of antibodies on the surface of these system would allow active targeting of drugs.

## 5 Conclusion

The findings reported in the present study demonstrated that PAR28-PEG-lipo can be more rapidly and easily modified with targeted antibody, in comparison with conventional methods. The prepared antibody-modified PAR28-PEG-lipo can be effectively taken up by cells depending on the targeted antibody. Based on these findings, PAR28-PEG-lipo demonstrates potential as a drug carrier that is capable of selective drug delivery to various cells by changing the targeting antibody modified on liposomes.

## Author contributions

Conceptualization, S.H. and K.K.; methodology, S.H.; validation, S.H.; formal analysis, S.H.; investigation, S.H., M.S. and S.I.; data curation, S.H.; writing—original draft preparation, S.H.; writing—review and editing, S.H., S.I., E.M. and K.K.; visualization, S.H.; supervision, S.H. and K.K; funding acquisition, S.H. All authors have read and agreed to the published version of the manuscript.

## Declaration of competing interest

The authors declare that they have no known competing financial interests or personal relationships that could have appeared to influence the work reported in this paper.
